# Duodenal Duplication Cyst having Ectopic Gastric and Pancreatic Tissues

**Published:** 2012-06-01

**Authors:** Binod Kumar Rai, Samina Zaman, Bilal Mirza, Ghazala Hanif, Afzal Sheikh

**Affiliations:** Department of Pediatric Surgery, The Children's Hospital and the Institute of Child Health Lahore, Pakistan; Department of Histopathology, The Children's Hospital and the Institute of Child Health Lahore, Pakistan; Department of Pediatric Surgery, The Children's Hospital and the Institute of Child Health Lahore, Pakistan; Department of Histopathology, The Children's Hospital and the Institute of Child Health Lahore, Pakistan; Department of Pediatric Surgery, The Children's Hospital and the Institute of Child Health Lahore, Pakistan

A 1-year-old female child presented with distention of abdomen, accompanied with occasional episodes of vomiting and abdominal pain for the past eight months with no history of constipation or fever. The child was vitally stable. On inspection upper abdomen was found distended and mild tenderness in epigastrium on deep palpation. Laboratory investigations were within normal limits. The plain radiograph of abdomen was unremarkable. Ultrasound scan showed a 6.9 cm x 7.5 cm sized cystic area with internal debris at porta hepatis, compressing the liver. CT scan showed a 5 cm x 7 cm sized cyst extending from porta hepatis to the duodenum (Fig. 1). The preoperative differentials were duodenal duplication and choledochal cyst.

**Figure:**
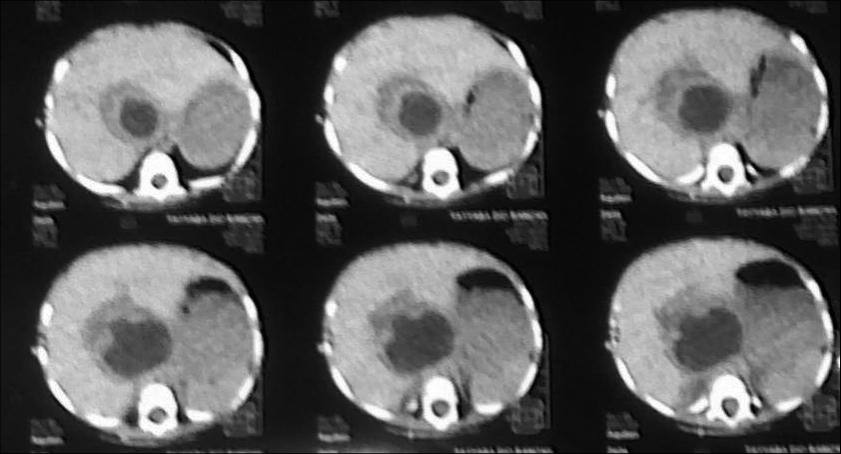
Figure 1: CT scan showing a hypo-dense area at porta hepatis.

At operation, a cyst medial to the gall bladder, pushing the stomach and the pancreas anteriorly and intimately related to the second part of the duodenum was found (Fig. 2).The content of the cyst was clear mucous on aspiration. Intra-operative cystogram was performed that ruled out its communication with biliary and alimentary tracts (Fig. 3). The wall of the cyst was opened and stripping of mucosal lining performed after excising resectable portion of the cyst. The cyst was sharing common wall with duodenum and was non - communicating. The child made an uneventful recovery and was discharged on the fifth postoperative day. Histopathology of the specimen showed gastric mucosal lined tissue having smooth muscles in the wall along with ectopic pancreatic tissue (Fig. 4,5).

**Figure:**
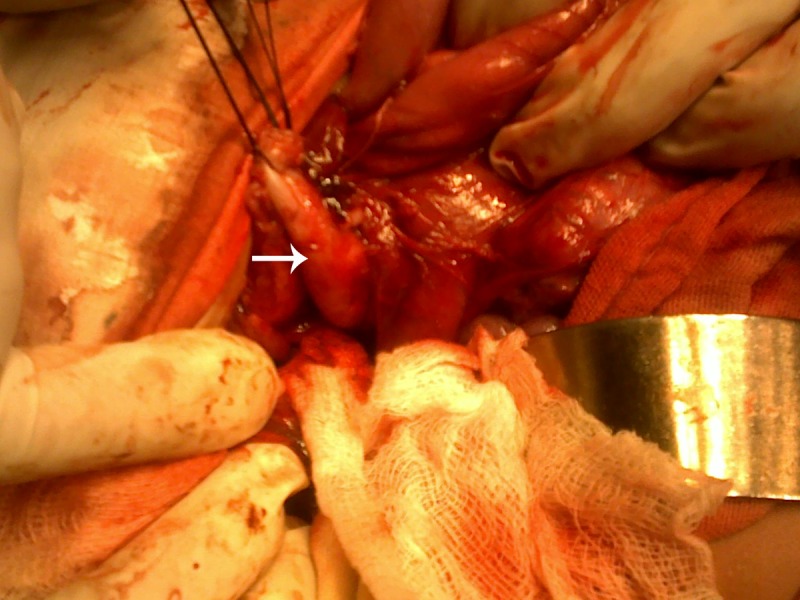
Figure 2: Dissection of the duodenal duplication cyst (Arrow).

**Figure:**
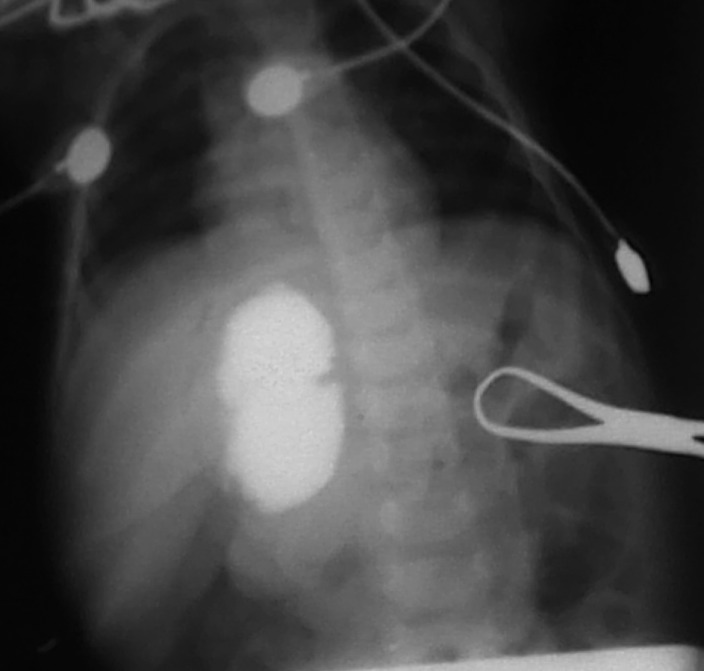
Figure 3: Intra-operative cystogram showing no communication with pancreatico-biliary ducts and duodenum.

**Figure:**
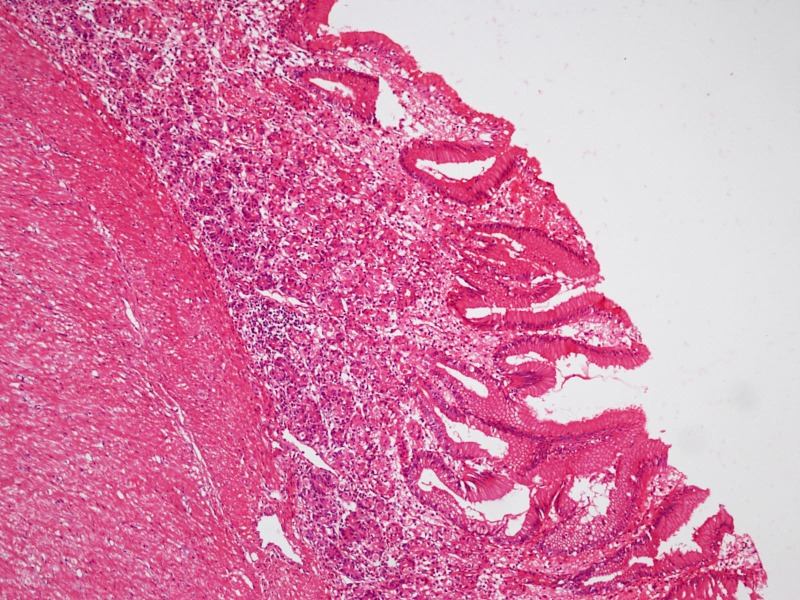
Figure 4: Microphotograph showing gastric mucosa with underlying muscle layer (x200).

**Figure:**
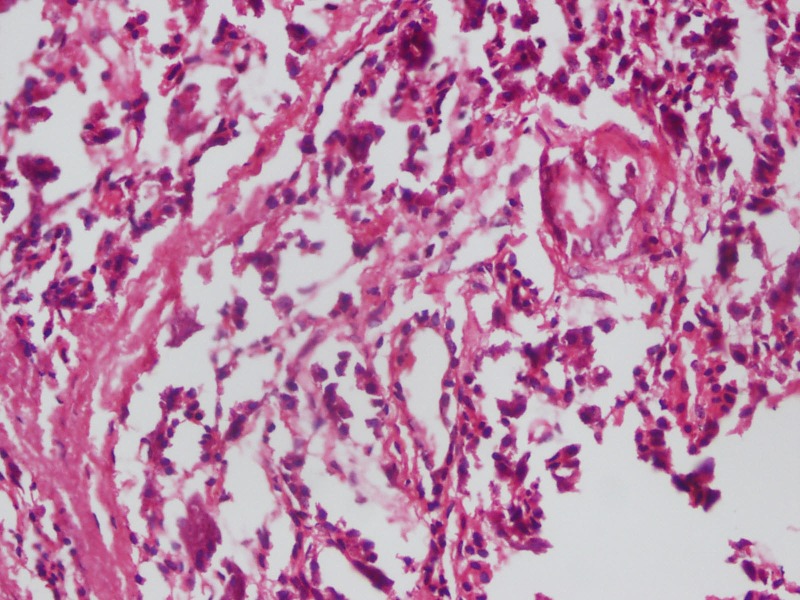
Figure 5: Microphotograph showing pancreatic tissue (x400).

## DISCUSSION

Gastrointestinal duplications may be cystic or tubular in shape with an intimate contact with the adjacent gut, smooth muscles in their wall, and mucosa resembling that of gastrointestinal tract. Duplications can present along any part of gastrointestinal tract, commonly along the ileum; duodenal duplications account for 5% of all gastrointestinal duplications. In 15-25% of cases ectopic gastric mucosa may be found. Few cases of duodenal duplications containing ectopic pancreatic tissue have been reported in literature. Concurrence of ectopic gastric and pancreatic tissues in a duodenal duplication cyst, as found in the index case, is however extremely rare [1-3]. 

Duodenal duplications may occur along the first and second parts of duodenum and are cystic with no communication with the intestinal lumen in most of the cases. Rarely, they can arise from pancreatico-biliary ducts. These cysts may be confused with choledochal cysts on account of their location between porta hepatis and duodenum. The presentation could be with abdominal pain, palpable epigastric mass, relapsing pancreatitis, and vomiting. In case of ectopic gastric mucosa, there could be intra-cystic hemorrhage or perforation of the cyst with peritonitis [2,3].

Ultrasound scan, upper gastrointestinal contrast study, CT scan, magnetic resonance cholangio pancreatography (MRCP), and endoscopy are important tools for preoperative diagnosis. Surgical resection is the treatment of choice for alimentary tract duplications. However, in case of duodenal duplications, excision of as much as part of duplication and mucosal stripping of the rest is preferred on account of its close proximity with pancreatico-biliary ductal systems. We proceeded on the same lines in our patient. Intra-operatic cystogram is mandatory to rule out its communication with pancreatico-biliary tree. Similarly, we have ruled out the communication of the cyst with pancreatico-biliary system and gut lumen by performing intra-operative cystogram. Drainage of the duplication cysts into the duodenum or into a Roux limb of jejunum is also an acceptable alternative [1-4]. 

## Footnotes

**Source of Support:** Nil

**Conflict of Interest:** None declared
